# Dr. Keats on Infirmary Discipline

**Published:** 1913-11-15

**Authors:** W. J. C. Keats

**Affiliations:** Medical Superintendent, Camberwell Infirmary


### PROBLEMS OF DISCIPLINE IN INFIRMARY WORK.

**Dr. W. J. C. KEATS, Medical Superintendent, Camberwell Infirmary, gives his Views.**

Nothing is truer in institutional life than to say that the rules and regulations necessary for its proper working, one though they be in aim, are conditioned by all sorts of factors peculiar to each institution itself. The structure, the design, the planning vary in each case as to details, and it is out of these details that the regulations grow. It is impossible, therefore, impartially to judge the value and importance, still less the character or harshness, of any such set of rules without a personal knowledge of the institution for which they were drafted. The public, however, represented mainly by the patients' friends, generally makes the test of its own case and comfort the exclusive test of the rules which regulate its own admission to and conduct in the institution; and nothing is easier, by suppressing the conditions which prevail, than for a man or woman on the staff with a grievance to gain ready sympathisers outside. The question of complaints is one in which the public always takes an interest, and one to which it mostly lends a ready ear, and the public ignorance concerning the conditions under which various regulations have been framed is balanced, sometimes to the disadvantage of the institution, by a knowledge on the part of the staff so complete and familiar by dint of practice that its members forget that what is the oldest or most trite matter to them is a difficulty laboriously to be explained to outsiders if the co-operation of these is to be sympathetically secured. In view of the recent controversy respecting the regulations at the Camberwell Infirmary, and their alleged arbitrary enforcement, our Commissioner visited the institution, and by the courtesy of Dr. William J. C. Keats, the medical superintendent, was shown over the institution, and was able to illustrate in the case of this infirmary the general principles enunciated above.

### The Site and Regulations.

"The whole question of administration here," Dr. Keats began, leading the way to the administration block, "is complicated by our restricted site. The architect, Mr. Edwin Hall, F.R.I.B.A., had to design an institution for over 800 beds, apart from staff accommodation, which includes 120 nurses' bedrooms on an area of about 5½ acres. The result is the plan cannot have full justice done to it, for, though the relation of the buildings and pavilions may be admired, their contiguity is an obvious disadvantage. Put shortly, the plan is something in the nature of a square, with, however, buildings in its centre. That creates, to take a simple but now notorious example, this sort of difficulty. Notwithstanding, I use the word with reluctance, for with loyal co-operation and common sense no difficulty should arise, or has indeed arisen in the whole course of my adminstrative experience here—and I have had fifteen years—until lately. On the right of the front entrance, in the administrative block, are the quarters of the assistant medical officers. These include a dining-room, in which is a piano, that they were permitted to hire on condition that it was never played except when all the doors leading from their quarters to those of night nurses and others asleep were carefully shut. A glance at the plan shows the necessity for such a rule. The inner side of the front block faces with verandah on to the Nurses' Home, one of the central buildings of which I spoke, and which, as you see, is only a few yards away. Just beyond the resident medical quarters are the wards. If the doors of these quarters are left open while the piano is playing the night nurses' sleep is disturbed, so is the rest of the patients in the wards. The delicate duty of the medical superintendent is therefore to do justice to all three—night nurses, patients, and the residents. That is the origin of this rule, which makes any breach of it a serious matter."

### Refractory Patients.

"Can I now see the convalescent wards?"

"Certainly. Our ward plan is to have a 24-bedded ward, a ward of four beds, and one of two beds, for observation or serious cases, and also a ward for six beds, which, as you will see, is used as a day room. In the block in which we are now the ward on the ground floor is for boys' surgical cases, and this is the ward in which the unruly cases occurred which have been so much discussed by outside scaremongers with the introduction of the word 'flogging' and the free use of phraseology of that kind. In a most impartial article which you published in The Hospital last week on 'Punishments in Poor-Law Infirmaries' you correctly pointed out that the position in regard to refractory patients in infirmaries is very different from that which prevails in the voluntary hospitals. The State institution cannot dismiss patients at a moment's notice, as a disciplinary measure. It cannot even turn out convalescing cases to make room for more serious ones."

### Boys in the Day-room.

"What is the actual position in regard to this particular ward?"

"The boys, as you see, are between the ages of five and sixteen. That is the refractory age. In the ward many are well enough, I am happy to say, to be playful, and, as a consequence of animal spirits and insufficient occupation, naughty. Why, an outsider may ask, can they not, whether ruly or unruly, be sent home? The answer, of course, is twofold. Under the most favourable conditions of home life, it would be doubtful medical policy to part with them; as it is, often they have no homes to go to at all! What happens is that when they are tired of jumping up and down on the bed springs, they begin to throw things, and when the throwing stage is reached it is a small step to breakages and destructiveness of all sorts. The boys mean no harm, but they do harm nevertheless. In the day room, for instance, they do—or did until I had to devise more drastic measures to stop them—chip off the paint, perhaps knock about the ceiling, and make themselves a nuisance with their noise. These pleasant signs of returning health cannot, however, be permitted to continue unchecked. Ordinary wear and tear of furniture, walls, floors, and utensils is quite a heavy enough item in repairs without its being added to in the spirit of mischief. Puck is a delightful person, but you cannot have him in an institution. Secondly, such conduct disorganises discipline and is generally demoralising to order, decorum, and routine. It is easy to laugh at such words as these, if you have no responsibility for an institution and the well-being of 1,000 persons; but one would have thought that every man and woman had some memories of his nursery days, or understood enough of the ordinary management of a house with children in it, to see that beyond a certain point mischievousness must stop.

"The question, then, is how to stop it. It is generally agreed, in fact it is invariably agreed, and put into practice, that conduct which is undesirable, after proper warning and explanation, must in lieu of these means be punished in some way. In common with other Poor-Law medical superintendents, corporal punishment is permitted to me in the case of children, but I am directly responsible for it, should it be inflicted, and that responsibility cannot be delegated. The class of boy-patient we have here is accustomed to the sight and infliction of such penalties in their own neighbourhood, if not in their own homes. It is the recognised way of saying 'No' finally, and for 'all.' But there are other equally ancient—I should have said equally common—and less drastic methods. You may stand a boy in a corner, you may do the equivalent of depriving him of cake at tea, or tart at dinner. In short, you may alter his diet. A Gilbertian situation arises, as your article pointed out, when a special or extra diet has been ordered and proves so beneficial that it excites a mischievousness which requires its modification in a privative sense. We should all be glad of further alternatives, but they are not forthcoming."

### Supervision and Extra Staff.

"Is it not suggested that a larger staff would provide extra supervision?"

"It may be suggested, but it is not the fact. The truth is that no staff, however large, could include persons who had no more duties than to watch for outbreaks of high spirits and to nip naughtiness in the bud. No staff is efficient unless each member has sufficient personal duties fully to occupy his or her working hours. The post of 'watcher' here hinted at will not bear examination."

"Another point is the question of visiting?"

"Take these wards, this institution. I believe no infirmary in the country is more completely open to public inspection than ours at Camberwell. The ordinary visiting days are Sundays and Thursdays, but if good reason can be shown a sympathetic ear is always given to special requests from patients' friends within the necessary limits of leaving ward work unembarrassed by the visitors. Every morning of the week the vestibule is crowded with incoming patients and their friends, and I have made a deliberate practice for many years now of giving up some time every morning to attending to the questions, inquiries, requests for advice, and what not, that are always made at this time. Except that the general public has no memory, and so never learns anything by experience, it would hardly be necessary to add, as your readers will mostly appreciate, that the question of visiting, as regards the risk of introducing infection, quite apart from that of carrying it away—were visits to infectious patients permitted without careful restriction—is always a serious and grave matter. Regulations about this, if about anything in the world, explain themselves. Yet capital has been made out of the fact that owing to the presence of infectious cases, the admissions had to be carefully controlled some time ago. If the process of visiting is a worrying, tiresome business to patients' friends, it is equally exacting on the superintendent and his staff."

### The Tennis Court Rules.

We had now left the administration block and passing along the covered corridor to the right in front of the Nurses' Home came upon an asphalt tennis court. Glancing at it Dr. Keats said: "As the whole controversy, as your readers will have been able to judge from the report of the committee that appeared in The Hospital last week, has its roots in trivial matters, the regulations concerning this tennis court give another example. First, note its situation in close proximity to the Nurses' Home, then study the regulations I have made concerning it. There being only one court, to which both the medical and nursing staff must have right of access, the rules state that the assistant medical officers may play every day between 2 p.m. and 5.30, that is to say, for three hours and a half. It must, of course, be understood that no desire to play entitles its indulgence if any of the residents have ward work to do. I found in practice, however, that two things were happening: first, that ward work was being neglected; and, secondly, that in order to finish a sett or begin one, as the case might be, work was being thrown upon nurses which, out of mistaken good nature and unselfishness, they had to complete in their off time.

### Impartiality to the Staff.

"Much as I welcomed this friendly spirit on the part of one section of staff towards another, it was impossible for me to sanction a custom which involved an interference with the prescribed duties of doctors and nurses respectively. The nurses' off time must be respected as jealously as that of the residents themselves. Now the simplicity, to hospital men the triteness, of these details is utterly unappreciated by the outside world, and if a malevolent person on hearing merely, say, that the residents' tennis had been curtailed chose to placard the borough with that bare statement—a malevolent person, however, would hit on a more telling phrase, I fancy—how easily an agitation, a petition signed not by 'Ratepayers and others,' but by 'ratepayers and Others,' could be concocted. These, however, are but illustrations of the simple fact that site and planning, apart from such considerations as the public safety in matters of infection, and the need for impartiality to all sections of the staff, affect and are responsible for the individual regulations which each separate institution has to draft and enforce. Fortunately, if recent events have enabled Camberwell Infirmary publicly to prove the need for the strict enforcement of disciplinary regulations, it can also give some hints of another kind to the institutional world."

[*Note.*—Owing to the exceptional pressure on our space, the extremely interesting "Hints on Practical Points" which follow have had to be held over to next week.—Ed.]

### REWARD FOR A MEDICAL WOMAN'S WORK.

The Lambeth Board of Guardians have recognised the services rendered at their schools at West Norwood by Dr. Alice N. V. Johnson, the medical superintendent, by increasing her salary from £200 to £220 per annum. When the new nursery is opened Dr. Johnson will receive a further £25 a year for administering to the needs of the babies. She has been in the service of the Board since 1904, and performs all minor operations on the patients. Fortunately the school is situated in one of the healthiest districts of South London, but with 600 children, many of them "in and outs," the labours of the medical superintendent are onerous. The difficulties are rendered more acute by the antiquated accoutrements. In the isolation block there was no bathroom, and patients were bathed in a portable utensil, which was wheeled in and out of the wards as it was required. The Board, however, has decided to replace it with up-to-date bathrooms.

